# Recanalization of occluded biliary intestinal anastomosis through utilizing percutaneous transhepatic cholangioscopy for a 2-year-old patient after liver transplantation

**DOI:** 10.1055/a-2384-9303

**Published:** 2024-09-04

**Authors:** Rui Chen, Jingyi Zhang, Tianhao Chen, Jie Zhang, Rongxing Zhou

**Affiliations:** 1 34753 Division of Biliary Surgery, Department of General Surgery, West China Hospital of Sichuan University, Chengdu, China; 2 34753 Research Center for Biliary Diseases, West China Hospital of Sichuan University, Chengdu, China; 3 34753 Department of Ultrasound, West China Hospital of Sichuan University, Chengdu, China

A 2-year-old girl with a history of liver transplantation for biliary atresia was admitted with recurrent fever and jaundice. Magnetic resonance cholangiopancreatography ( Fig. 1 ) showed a stricture of the bilioenteric anastomosis. Two attempts at percutaneous transhepatic cholangiography drainage (PTCD) failed ( Fig. 2 ). Percutaneous transhepatic cholangioscopy (PTCS) was therefore applied to provide direct visualization of any intraductal lesions or confirm a benign stricture, which is generally observed as having a smooth mucosal surface, tapered luminal narrowing, short stricture segment, and the absence of neovascularization 1 ( Video 1 ).

**Fig. 1:**
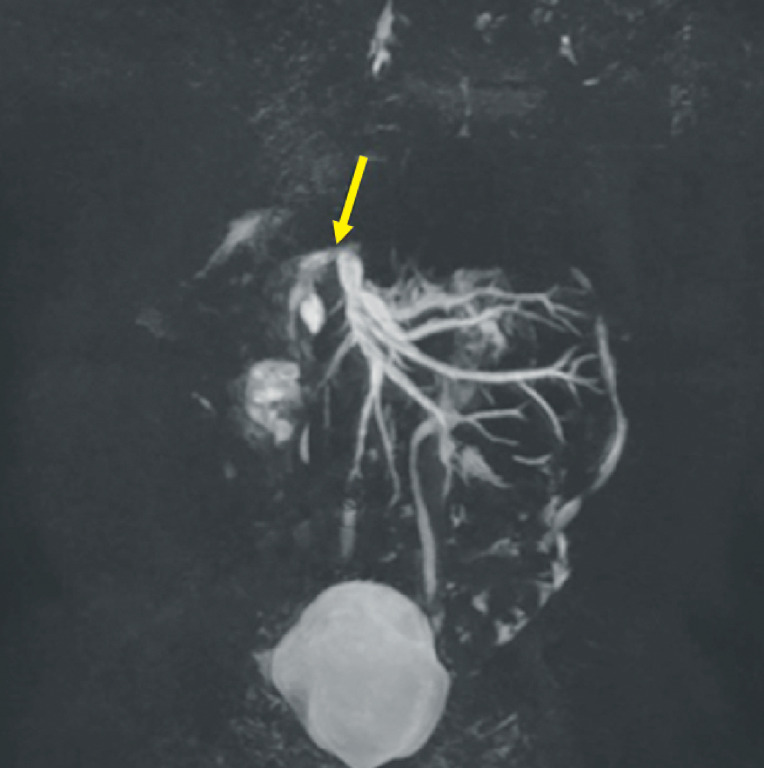
Magnetic resonance cholangiopancreatography showed a stricture of the biliary intestinal anastomosis (yellow arrow).

**Fig. 2:**
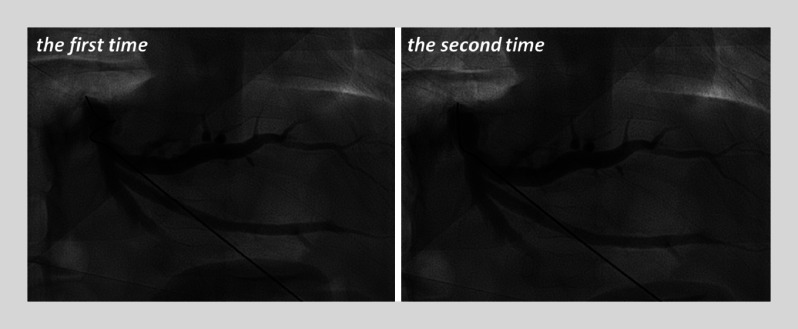
In both percutaneous transhepatic cholangiography drainage (PTCD) procedures, the guidewire failed to pass through the anastomosis due to severe narrowing.

The recanalization of occluded biliary intestinal anastomosis was successfully performed under the direct visualization of percutaneous transhepatic cholangioscopy (PTCS) for the 2-year-old patient after failed percutaneous transhepatic cholangiography drainage (PTCD).Video 1

To minimize the patient’s radiation dose exposure, ultrasound was used instead of interventional radiography to evaluate the intrahepatic bile ducts. The guidewire was inserted through the PTCD sinus, followed by sequential dilation of the tract until a 16-Fr sheath could be placed. A rigid cholangioscope was inserted under the guidance of the guidewire, and the completely obstructed bilio-enteric anastomotic site was then directly visualized after clearing the hepatolithiasis ( Fig. 3 ). The guidewire was passed through the anastomosis after repeated attempts. Balloon dilation was performed repeatedly until significant dilation of the anastomosis was achieved ( Fig. 4 ). The intraoperative cholangiography confirmed the disappearance of the occluded biliary intestinal anastomosis, with good transition of contrast medium from bile ducts to bowel ( Fig. 5 ). Finally, a 16-Fr bile drainage tube was placed through the anastomosis to support and drain. There were no postoperative complications and the patient was discharged 3 days after surgery.

**Fig. 3:**
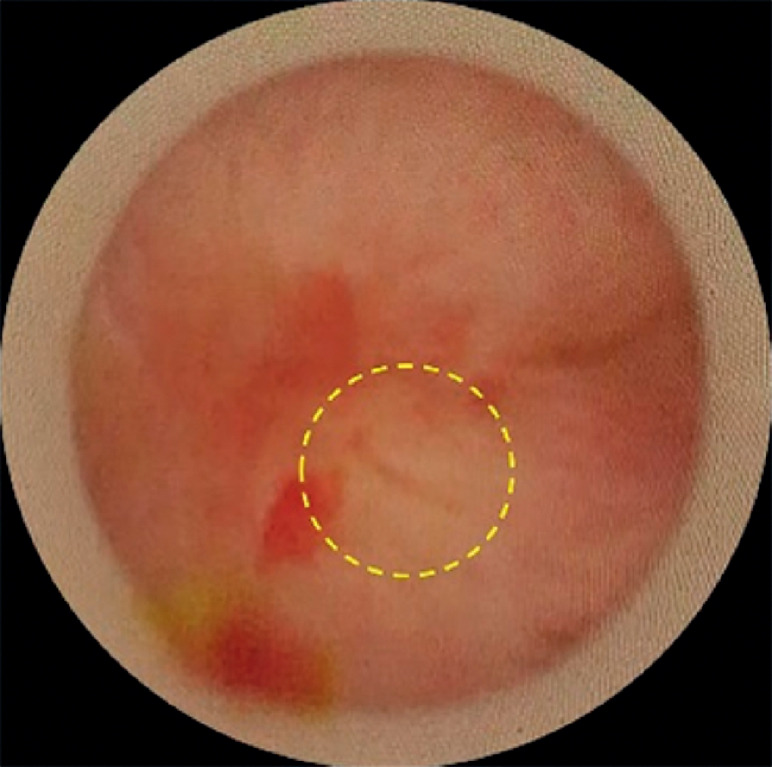
The obstructed anastomosis (yellow dashed circle) was observed under the direct visualization of the rigid cholangioscope.

**Fig. 4:**
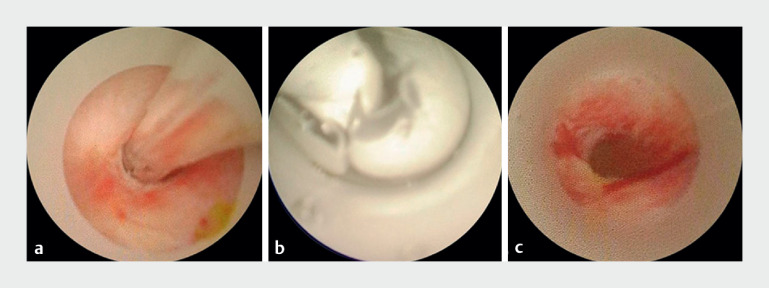
The balloon was used to repeatedly dilate the narrow tracts until there was a noticeable dilation at the anastomotic site. **a** The balloon was placed through the anastomosis. **b** The pressure of the balloon was maintained at 8 bar for 1 minute for continuous expansion. **c** The originally narrow anastomosis showed significant dilation after multiple expansions.

**Fig. 5:**
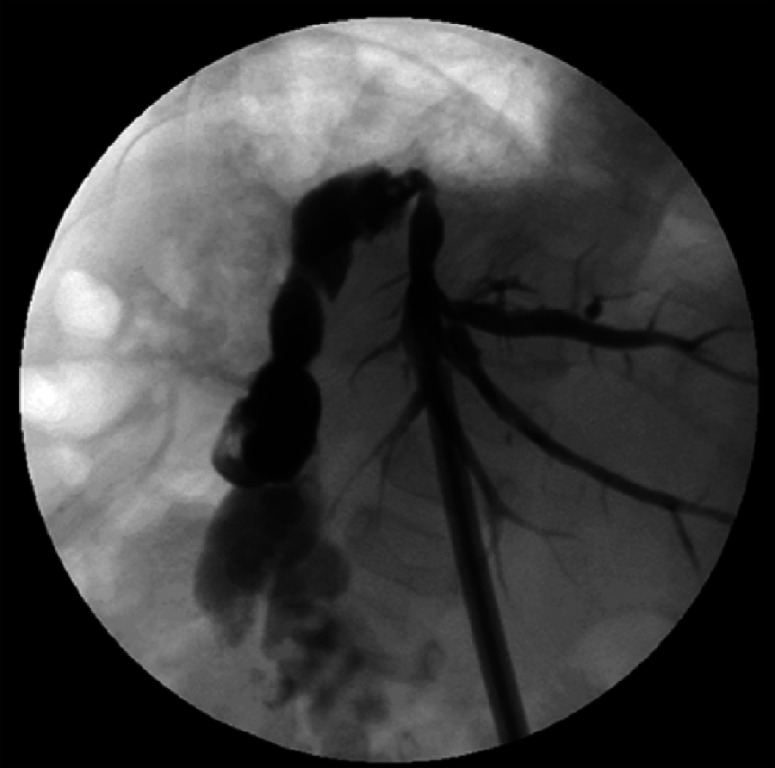
The intraoperative cholangiography confirmed the disappearance of the occluded biliary intestinal anastomosis, with good transition of contrast medium from bile ducts to bowel.

Anastomotic stricture is a common complication after liver transplantation 2 . PTCD has become the preferred choice for most patients given the changes in anatomical structure 3 . Compared with PTCD, PTCS has the advantages of operating under direct vision and reducing the radiation exposure 4 , and it has been widely used in adults with biliary-enteric anastomotic strictures 5 . This study demonstrated that PTCS might also be a safe and feasible minimally invasive treatment for anastomotic stricture following pediatric liver transplantation.

Endoscopy_UCTN_Code_TTT_1AR_2AG

## Notes

Endoscopy E-Videos https://eref.thieme.de/e-videos
*E-Videos* is an open access online section of the journal *Endoscopy* , reporting on interesting cases and new techniques in gastroenterological endoscopy. All papers include a high-quality video and are published with a Creative Commons CC-BY license. Endoscopy E-Videos qualify for HINARI discounts and waivers and eligibility is automatically checked during the submission process. We grant 100% waivers to articles whose corresponding authors are based in Group A countries and 50% waivers to those who are based in Group B countries as classified by Research4Life (see: https://www.research4life.org/access/eligibility/ ). This section has its own submission website at https://mc.manuscriptcentral.com/e-videos .
